# Revealing Neurocomputational Mechanisms of Reinforcement Learning and Decision-Making With the hBayesDM Package

**DOI:** 10.1162/CPSY_a_00002

**Published:** 2017-10-01

**Authors:** Woo-Young Ahn, Nathaniel Haines, Lei Zhang

**Affiliations:** 1 Department of Psychology, The Ohio State University, Columbus, OH; 2 Institute for Systems Neuroscience, University Medical Center Hamburg-Eppendorf, Hamburg, Germany

**Keywords:** reinforcement learning, decision-making, hierarchical Bayesian modeling, model-based fMRI

## Abstract

Reinforcement learning and decision-making (RLDM) provide a quantitative framework and computational theories with which we can disentangle psychiatric conditions into the basic dimensions of neurocognitive functioning. RLDM offer a novel approach to assessing and potentially diagnosing psychiatric patients, and there is growing enthusiasm for both RLDM and computational psychiatry among clinical researchers. Such a framework can also provide insights into the brain substrates of particular RLDM processes, as exemplified by model-based analysis of data from functional magnetic resonance imaging (fMRI) or electroencephalography (EEG). However, researchers often find the approach too technical and have difficulty adopting it for their research. Thus, a critical need remains to develop a user-friendly tool for the wide dissemination of computational psychiatric methods. We introduce an R package called hBayesDM (hierarchical Bayesian modeling of Decision-Making tasks), which offers computational modeling of an array of RLDM tasks and social exchange games. The hBayesDM package offers state-of-the-art hierarchical Bayesian modeling, in which both individual and group parameters (i.e., posterior distributions) are estimated simultaneously in a mutually constraining fashion. At the same time, the package is extremely user-friendly: users can perform computational modeling, output visualization, and Bayesian model comparisons, each with a single line of coding. Users can also extract the trial-by-trial latent variables (e.g., prediction errors) required for model-based fMRI/EEG. With the hBayesDM package, we anticipate that anyone with minimal knowledge of programming can take advantage of cutting-edge computational-modeling approaches to investigate the underlying processes of and interactions between multiple decision-making (e.g., goal-directed, habitual, and Pavlovian) systems. In this way, we expect that the hBayesDM package will contribute to the dissemination of advanced modeling approaches and enable a wide range of researchers to easily perform computational psychiatric research within different populations.

## INTRODUCTION

**Figure 1.**
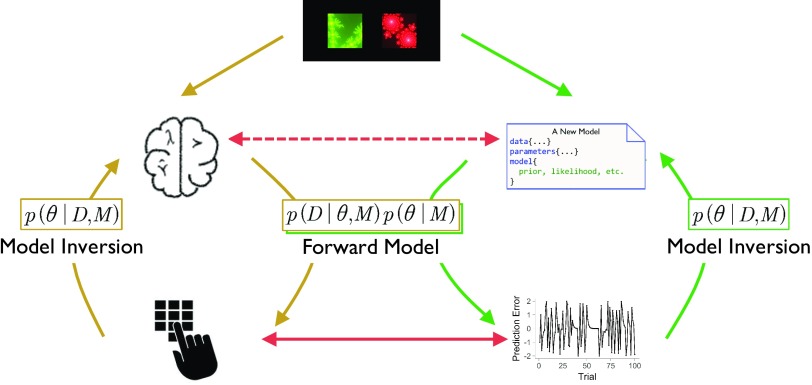
**Conceptual schema of computational modeling.** Starting with a certain RLDM paradigm, the left pathway (yellow arrows) represents that the human brain produces behavioral responses (forward model) that we observe and measure. These observed outcomes can be used to make inferences about cognitive mechanisms (model inversion), but oftentimes this is difficult to achieve. One solution is to build cognitive models (green arrows) that produce predictions (forward model) and can also be inferred on the basis of those predictions (model inversion). As such, we are able to approximate brain mechanisms (dashed red line) by directly linking the model predictions (e.g., reward prediction error) with the observed outcomes (solid red line).

*Computational modeling* (a.k.a. *cognitive modeling*) describes human information processing in terms of basic principles of cognition, which are defined in formal mathematical notation (Figure 1). Unlike verbalized or conceptualized approaches, computational modeling has the merit of allowing researchers to generate precise predictions and quantitatively test competing hypotheses (Busemeyer & Diederich, 2010; Forstmann & Wagenmakers, 2015; Lewandowsky & Farrell, 2010). Computational modeling has been particularly useful in the reinforcement-learning and decision-making (RLDM) fields (Dayan & Daw, 2008; Rangel, Camerer, & Montague, 2008); it has also been integrated into the analysis of neural data, including data from functional magnetic resonance imaging (fMRI) and electroencephalography (EEG) (e.g., (Cavanagh, Eisenberg, Guitart-Masip, Huys, & Frank, 2013; Daw, O’Doherty, Dayan, Seymour, & Dolan, 2006; Gläscher, Hampton, & O’Doherty, 2009; Hampton, Bossaerts, & O’Doherty, 2006; Iglesias et al., 2013; Li, Schiller, Schoenbaum, Phelps, & Daw, 2011; Mars et al., 2008; O’Doherty et al., 2004; O’Doherty, Hampton, & Kim, 2007; Xiang, Lohrenz, & Montague, 2013).

As has been summarized in recent review articles (Ahn & Busemeyer, 2016; Friston, Stephan, Montague, & Dolan, 2014; Huys, Maia, & Frank, 2016; Montague, Dolan, Friston, & Dayan, 2012; Stephan, Bach, et al., 2016; Stephan, Binder, et al., 2016; Stephan, Iglesias, Heinzle, & Diaconescu, 2015; Wang & Krystal, 2014; Wiecki, Poland, & Frank, 2015), computational modeling has gained much attention for its usefulness in investigating psychiatric conditions. Exemplified by the Research Domain Criteria (RDoC; Insel, 2014) and precision medicine, a growing consensus is advocating that diagnosis and treatment decisions incorporate the underlying cognitive and neurobiological underpinnings of psychiatric conditions, instead of relying only on behavioral symptoms. Toward this end, a new field, called *computational psychiatry* (e.g., Friston et al., 2014; Montague et al., 2012), aims to discover the neurocognitive mechanisms underlying normal and abnormal conditions by combining cutting-edge neurobiological and computational tools.

Performing computational psychiatric research, however—especially computational modeling—is a challenging task for many clinical researchers or those with limited quantitative skills. Computational modeling involves multiple steps, including designing/ adopting laboratory tasks, building a theoretical framework of the task in terms of a set of assumptions and mathematical equations, formulating multiple computational models based on the assumptions, estimating the parameters of each model, and quantitatively comparing the models of interest (e.g., Busemeyer & Diederich, 2010; Wiecki et al., 2015). Thus, a pressing issue is how to train clinical researchers in mental health (e.g., psychiatrists and clinical psychologists) so that they can receive in-depth training across several related fields, including cognitive science, advanced statistics, and neuroscience (Montague et al., 2012). For the dissemination of computational psychiatry, we believe that a critical need to develop user-friendly tools for computational modeling still remains. In fact, several software packages for this purpose do exist, but most of them focus on only a single class of modeling, such as sequential-sampling models (Matzke et al., 2013; Singmann et al., 2016; Vincent, 2016; Wabersich & Vandekerckhove, 2014; Wiecki, Sofer, & Frank, 2013). An exception is the Variational Bayesian Analysis (VBA) MATLAB toolbox (Daunizeau, Adam, & Rigoux, 2014), which allows users to fit and compare various models using variational Bayesian algorithms. However, we believe users will still need some amount of programming skill and a background in computational modeling in order to model various tasks with the VBA toolbox.

In this article, we describe a free R package, *hBayesDM* (for “hierarchical Bayesian modeling of Decision-Making tasks”), which we developed for the dissemination of computational modeling to a wide range of researchers. The hBayesDM package offers hierarchical Bayesian analysis (HBA; see the Mathematical Formulation of Hierarchical Bayesian Models section for more details) of various computational models for an array of decision-making tasks (see Table 1 for a list of the tasks and models currently available). With the user-friendly hBayesDM package, users can perform model fitting with HBA, output visualization, and model comparisons—*each with a single line of coding*. Example datasets are also available to make it easy to use hBayesDM. With the package, users can extract the trial-by-trial latent vari ables (e.g., prediction errors) that are required for model-based fMRI/EEG (see the Extracting Trial-by-Trial Regressors for Model-Based fMRI/EEG Analysis section). Experienced users can even develop new models based on our framework and codes. All source codes are publically available at our GitHub repository (https://github.com/ccs-lab/hBayesDM). Users also can post questions to our mailing list (https://groups.google.com/forum/#!forum/hbayesdm-users) or make suggestions by posting new issues to the GitHub repository. By making all steps for computational modeling user-friendly, we hope the hBayesDM package will allow even researchers with minimal programming knowledge to perform computational psychiatric research.

**Table 1.** List of the tasks and models currently (as of version 0.3.0) implemented in the hBayesDM package

**Task (Alpha Order) as of Version 0.3.0**	**Required Columns in the Data File**	**Model Names**	**hBayesDM Functions**	**Numbers of Parameters (per Subject)**	**References**
Delay discounting	**subjID**: subject identifier	Constant-sensitivity	dd_cs	3	Ebert & Prelec (2007)
	**delay_later**: time delay of	Exponential	dd_exp	2	Samuelson (1937)
	later option	Hyperbolic	dd_hyperbolic	2	Mazur (1987)
	**amount_later:** amount of				
	later option				
	**delay_sooner:** time delay of				
	sooner option				
	**amount_later:** amount of				
	sooner option				
	**choice:** chosen option				
Iowa gambling	**subjID:** subject identifier	Prospect valence	igt_pvl_decay	4	Ahn et al. (2014),
	**choice:** chosen deck	learning-DecayRI	igt_pvl_delta	4	Ahn et al. 2011
	(= 1, 2, 3, or 4)	Prospect valence	igt_vpp	7	Ahn et al. (2008)
	**gain:** amount gained	learning-Delta			Worthy, Pang, & Byrne (2013)
	on each trial	Value-plus-			
	**loss:** amount lost on	perseverance			
	each trial				
Orthogonalized	**subjID:**subject identifier	RW + noise	gng_m1	3	Guitart-Masip et al. (2012)
go/no-go	**cue:** cue number (= 1, 2,	RW + noise + go bias	gng_m2	4	
	3, or 4)	RW + noise + go bias +	gng_m3	5	"
	**keyPressed:** pressed (1) or	Pav. bias	gng_m4	6	"
	not (0)	RW(rew/pun) + noise +			Cavanagh et al. (2013)
	**outcome:** outcome on each	go bias + Pav. bias			
	trial (= − 1, 0, or 1)				
Probabilistic reversal	**subjID:** subject identifier	Experience-weighted	prl_ewa	3	den Ouden et al. (2013)
learning	**choice:** chosen option (= 1	attraction	prl_fictitious	3	
	or 2)	Fictitious update	prl_rp	3	Gläscher, Hampton, & ODoherty (2009)
	**outcome:** reward (1) or	Reward–punishment			
	loss (− 1) on each trial				den Ouden et al. (2013)
Risk aversion	**subjID:** subject identifier	Prospect theory (PT)	ra_prospect	3	Sokol-Hessner et al. (2009)
	**gain:** possible (50%) gain	PT without loss	ra_noLA	2	
	of risky option	aversion (LA)	ra_noRA	2	Tom, Fox, Trepel, & Poldrack (2007)
	**loss:** possible (50%) loss	PT without risk			
	of risky option	aversion (RA)			
	**cert:** certain option				
	**gamble:** gamble was taken				
	(= 1) or not (= 0)				
Two-armed bandit	**subjID:** subject identifier	Rescorla–Wagner	bandit2arm_delta	2	Erev et al et al. (2010)
	**choice:** chosen option (= 1	(delta)			Hertwig, Barron, Weber, & Erev (2004)
	or 2)				
	**outcome:** outcome on each				
	trial				

Ultimatum game	**subjID:** subject identifier	Ideal Bayesian observer	ug_bayes	3	Xiang et al. (2013)
	**offer:** offer made by	Rescorla–Wagner (delta)	ug_delta	3	Gu et al. (2015)
	proposer				
	**accept:** accepted (1) or				
	declined (0) by responder				

The remainder of this article is organized as follows. First, we describe each of the tasks and models currently implemented in the hBayesDM package (see Tasks and Computational Models Implemented in hBayesDM section). Second, we briefly describe HBA and why we adopted it for computational modeling (see Mathematical Formulation of Hierarchical Bayesian Models section). Third, we provide a detailed mathematical formulation of hierarchical Bayesian models (see Performing Hierarchial Bayesian Analysis With Stan section). Fourth, we provide step-by-step tutorials on how to use the hBayesDM package (see Step-by-Step Tutorials for the hBayesDM Package section). Finally, we discuss future directions and potential limitations of the package (see Future Directions). Readers who are not interested in the technical details may skip Mathematical Formulation of Hierarchical Bayesian Models and the equations in Performing Hierarchial Bayesian Analysis With Stan.

## TASKS AND COMPUTATIONAL MODELS IMPLEMENTED IN hBayesDM

Table 1 shows the list of tasks and computational models currently implemented in the hBayesDM package (as of version 0.3.0). Note that some tasks have multiple computational models and that users can compare model performance within the hBayesDM framework (see Step-by-Step Tutorials for the hBayesDM Package). To fit models to a task, first the user must prepare trial-by-trial data as a text file (*.txt) in which each row (observation) contains the columns required for the given task (see Table 1). Users can also use each task’s sample dataset as a template.

Below, we describe each task and its computational model(s), briefly review its applications to healthy and clinical populations, and describe the model parameters. For brevity, we refer readers to original articles for the full details of the experimental design and computational models, and to the package help files for example codes that detail how to estimate and extract the parameters from each model. The package help files can be found by issuing the following command within the R console:

?hBayesDM

The command above will open the main help page, from which one can then navigate to the corresponding task/model. Users can also directly look up a help file for each task/model by calling its help file, which follows the form ?function_name (e.g., ?dd_cs; see Table 1 for a list of these functions). Each help file provides working codes to run a concrete real-data example from start to finish.

### The Delay-Discounting Task

The delay-discounting task (DDT; Rachlin, Raineri, & Cross, 1991) is designed to estimate how much an individual discounts temporally delayed larger outcomes in comparison to smaller–sooner ones. On each trial of the DDT, two options are presented: a sooner and smaller reward (e.g., $5 now) and a later and larger reward (e.g., $20 next week). Subjects are asked to choose which option they prefer on each trial.

The DDT has been widely studied in healthy populations (e.g., Green & Myerson, 2004; Kable & Glimcher, 2007) and delay discounting has been associated with cognitive abilities such as intelligence (Shamosh et al., 2008) and working memory (Hinson, Jameson, & Whitney, 2003). Steeper delay discounting is a strong behavioral marker for addictive behaviors (Ahn, Ramesh, Moeller, & Vassileva, 2016; Ahn & Vassileva, 2016; Bickel, 2015; Green & Myerson, 2004; MacKillop, 2013) and has also been associated with other psychiatric conditions, including schizophrenia (Ahn, Rass, et al., 2011; Heerey, Matveeva, & Gold, 2011; Heerey, Robinson, McMahon, & Gold, 2007) and bipolar disorder (Ahn, Rass, et al., 2011). The hBayesDM package currently contains three different models for the DDT:

1.  dd_cs (constant-sensitivity model; Ebert & Prelec, 2007)
  Exponential discounting rate (0 <*r* < 1)
  Impatience (0 < *s* <10)
  Inverse temperature (0 < *β*< 5)
2.  dd_exp (exponential model; Samuelson, 1937)
  Exponential discounting rate (0 < *r* < 1)
  Inverse temperature (0 < *β* < 5)
3.  dd_hyperbolic (hyperbolic model; Mazur, 1987)
  Discounting rate (0 < *k* < 1)
  Inverse temperature (0 < *β* < 5)

#### DDT: Parameter descriptions

In the exponential and hyperbolic models, temporal discounting of future (i.e., delayed) rewards is described by a single parameter, the discounting rate (0 < *r* < 1), which indicates how much future rewards are discounted. High and low discounting rates reflect greater and lesser discounting of future rewards, respectively. In the exponential and hyperbolic models, the value of a delayed reward is discounted in an exponential and hyperbolic form, respectively. The constant-sensitivity (CS) model has an additional parame ter, called *time sensitivity* (0 < *s* < 10). When *s* is equal to 1, the CS model reduces to the ex ponential model. Values of *s* near 0 lead to a simple “present–future dichotomy” in which all future rewards are steeply discounted to a certain subjective value, irrespective of delays. Values of *s* greater than 1 result in an “extended-present” heuristic, in which rewards during the extended present are valued nearly equally, and future rewards outside the extended present have zero value.

All models use the softmax choice rule with an inverse-temperature parameter (Kaelbling, Littman, & Moore, 1996; Luce, 1959), which reflects how deterministically individuals’ choices are made with respect to the strength (subjective value) of the alternative choices. High and low inverse temperatures represent more deterministic and more random choices, respectively.

### The Iowa Gambling Task

The Iowa Gambling Task (IGT; Bechara, Damasio, Damasio, & Anderson, 1994) was originally developed to assess decision-making deficits of patients with ventromedial prefrontal cortex lesions. On each trial, subjects are presented with four decks of cards. Two decks are advantageous (good) and the other two decks disadvantageous (bad), in terms of long-term gains. Subjects are instructed to choose decks that maximize long-term gains, which they are expected to learn by trial and error. From a statistical perspective, the IGT is a four-armed bandit problem.

The IGT has been used extensively to study decision-making in several psychiatric populations (Ahn et al., 2014; Bechara & Martin, 2004; Bechara et al., 2001; Bolla et al., 2003; Grant, Contoreggi, & London, 2000; Vassileva, Gonzalez, Bechara, & Martin, 2007). The hBayesDM package currently contains three different models for the IGT:

1.  igt_pvl_decay (Ahn et al., 2014; Ahn, Krawitz, Kim, Busemeyer, & Brown, 2011)
  Decay rate (0 < *A* < 1)
  Shape (0 < *α* < 2)
  Consistency (0 < *c* < 5)
  Loss aversion (0 < *λ* < 10)
2.  igt_pvl_delta (Ahn, Busemeyer, Wagenmakers, & Stout, 2008)
  Learning rate (0 < *A* < 1)
  Shape (0 < *α* < 2)
  Consistency (0 < *c* < 5)
  Loss aversion (0 < *λ* < 10)
3.  igt_vpp (Worthy, Pang, & Byrne, 2013)
  Learning rate (0 < *A* < 1)
  Shape (0 < *α* < 2)
  Consistency (0 < *c* < 5)
  Loss aversion (0 < *λ* < 10)
  Perseverance gain impact ($-\infty <\epsilon_{p}<\infty$)
  Perseverance loss impact ($-\infty <\epsilon_{n}<\infty$)
  Perseverance decay rate (0 < *k* < 1)
  Reinforcement-learning weight (0 < *ω* < 1)

#### IGT: Parameter descriptions

The Prospect Valence Learning (PVL) model with delta rule (PVL-delta) uses a Rescorla–Wagner updating equation (Rescorla & Wagner, 1972) to update the expected value of the selected deck on each trial. The expected value is updated with a learning rate parameter (0 < *A* < 1) and a prediction error term, where *A* close to 1 places more weight on recent outcomes, and *A* close to 0 places more weight on past outcomes; the prediction error is the difference between the predicted and experienced outcomes. The shape (0 < *α* < 2) and loss aversion (0 < *λ* < 1) parameters control the shape of the utility (power) function and the effect of losses relative to gains, respectively. Values of *α* greater than 1 indicate that the utility of an outcome is convex, and values less than 1 indicate that the utility is concave. Values of *λ* greater than or less than 1 indicate greater or reduced sensitivity, respectively, to losses relative to gains. The consistency parameter (0 < *c* < 1) is an inverse-temperature parameter (refer to The Delay-Discounting Task for details).

The PVL model with decay rule (PVL-decay) uses the same shape, loss aversion, and consistency parameters as the PVL-delta, but a recency parameter (0 < *A* < 1) is used for value updating. The recency parameter indicates how much the expected values of all decks are discounted on each trial.

The PVL-delta model is nested within the Value-Plus-Perseverance (VPP) model, which is a hybrid model of PVL-delta and a heuristic strategy of perseverance. The perseverance decay rate (0 < *k* < 1) decays the perseverance strengths of all choices on each trial, akin to how PVL-decay’s recency parameter affects the expected value. The parameters for the impacts of gain ($-\infty <\epsilon_{p}<\infty$) and loss ($-\infty <\epsilon_{n}<\infty$) on perseverance reflect how the perseverance value changes after wins and losses, respectively; positive values reflect a tendency to make the same choice, and negative values a tendency to switch choices. The reinforcement-learning weight (0 < *ω* < 1) is a mixing parameter that controls how much decision weight is given to the reinforcement-learning versus the perseverance term. High versus low values reflect more versus less reliance on the reinforcement-learning term, respectively.

### The Orthogonalized Go/No-Go Task

Animals use Pavlovian and instrumental controllers when taking action. The Pavlovian controller selects approaching/engaging actions with predictors of appetitive outcomes or avoiding/inhibiting actions with predictors of aversive outcomes. The instrumental controller, on the other hand, selects actions on the basis of the action–outcome contingencies of the environment. The two controllers typically cooperate, but sometimes they compete with each other (e.g., Dayan, Niv, Seymour, & Daw, 2006). The orthogonalized go/no-go (GNG) task (Guitart-Masip et al., 2012) is designed to examine the interaction between the two controllers by orthogonalizing the action requirement (go vs. no go) versus the valence of the outcome (winning vs. avoiding losing money).

Each trial of the orthogonal GNG task has three events in the following sequence: cue presentation, target detection, and outcome presentation. First, one of four cues is presented (“Go to win,” “Go to avoid (losing),” “NoGo to win,” or “NoGo to avoid”). After some delay, a target (“circle”) is presented on the screen, and subjects need to respond with either a *go* (press a button) or *no go* (withhold the button press). Then subjects receive a probabilistic (e.g., 80%) outcome. See Guitart-Masip et al. (2012) for more details of the experimental design.

The orthogonalized GNG task has been used to study decision-making in healthy populations (Cavanagh et al., 2013), age-related changes in midbrain structural integrity in older adults (Chowdhury, Guitart-Masip, Lambert, Dolan, & Duzel, 2013), and negative symptoms of schizophrenia (Albrecht, Waltz, Cavanagh, Frank, & Gold, 2016). The interaction between Pavlovian and instrumental controllers might also play a role in addiction problems (Guitart-Masip, Duzel, Dolan, & Dayan, 2014). The hBayesDM package currently contains four different models for the orthogonalized GNG task:

1.  gng_m1 (M1 in Guitart-Masip et al., 2012)
  Lapse rate (0 < *ξ* < 1)
  Learning rate (0 < *ϵ* < 1)
  Effective size of a reinforcement (0<ρ<∞)
2.  gng_m2 (M2 in Guitart-Masip et al., 2012)
  Lapse rate (0 < *ξ* < 1)
  Learning rate (0 < *ϵ* < 1)
  Go bias (−∞<b<∞)
  Effective size of a reinforcement (0<ρ<∞)
3.  gng_m3 (M3 in Guitart-Masip et al., 2012)
  Lapse rate (0 < *ξ* < 1)
  Learning rate (0 < *ϵ* < 1)
  Go bias (−∞<b<∞)
  Pavlovian bias (−∞<π<∞)
  Effective size of a reinforcement (0<ρ<∞)
4.  gng_m4 (M5 in Cavanagh et al., 2013)
  Lapse rate (0 < *ξ* < 1)
  Learning rate (0 < *ϵ* < 1)
  Go bias (−∞<b<∞)
  Pavlovian bias (−∞<π<∞)
  Effective size of reward reinforcement ($0<\rho_{rew}<\infty$)
  Effective size of punishment reinforcement ($0<\rho_{pun}<\infty$)

#### GNG: Parameter descriptions

All models for the GNG task include a lapse rate parameter (0 < *ξ* < 1), a learning rate parameter (0 < *ϵ* < 1; refer to IGT: Parameter descriptions for details), and a parameter for the effective size of reinforcement (0<ρ<∞). The lapse rate parameter captures the proportion of random choices made, regardless of the strength of their action probabilities. The *ρ* parameter determines the effective size of a reinforcement. The gng_m4 model has separate effective size parameters for reward ($0<\rho_{rew}<\infty$) and punishment ($0<\rho_{pun}<\infty$), allowing for rewards and punishments to be evaluated differently.

Three GNG models (gng_m2, gng_m3, and gng_m4) include a go bias parameter (−∞<b<∞). Go bias reflects a tendency to respond (*go*), regardless of the action–outcome associations; high or low values for *b* reflect a high or a low tendency to make a go (motor) response, respectively.

Two GNG models (gng_m3 and gng_m4) include a Pavlovian bias parameter (−∞<π<∞). Pavlovian bias reflects a tendency to make responses that are Pavlovian congruent: that is, to promote or inhibit *go*if the expected value of the stimulus is positive (appetitive) or negative (aversive), respectively.

### Probabilistic Reversal-Learning Task

Environments often have higher-order structures, such as interdependencies between the stimuli, actions, and outcomes. In such environments, subjects need to infer and make use of the structures in order to make optimal decisions. In the probabilistic reversal-learning (PRL) task, higher-order structure exists such that the reward distributions of two stimuli are anticorrelated (e.g., if one option has a reward rate of 80%, the other option has a reward rate of [100 – 80]%, which is 20%). Subjects need to learn the higher-order structure and take it into account to optimize their decision-making and to maximize earnings.

In a typical PRL task, two stimuli are presented to a subject. The choice of a “correct” or good stimulus will usually lead to a monetary gain (e.g., 70%), whereas the choice of an “incorrect” or bad stimulus will usually lead to a monetary loss. The reward contingencies will reverse at fixed points (e.g., Murphy, Michael, Robbins, & Sahakian, 2003) or will be triggered by consecutive correct choices (Cools, Clark, Owen, & Robbins, 2002; Hampton et al., 2006).

The PRL task has been widely used to study reversal learning in healthy individuals (Cools et al., 2002; den Ouden et al., 2013; Gläscher et al., 2009). The PRL has been also used to study decision-making deficits associated with prefrontal cortex lesions (e.g., Fellows & Farah, 2003; Rolls, Hornak, Wade, & McGrath, 1994), as well as Parkinson’s disease (e.g., Cools, Lewis, Clark, Barker, & Robbins, 2007; Swainson et al., 2000), schizophrenia (e.g., Waltz & Gold, 2007), and cocaine dependence (Ersche, Roiser, Robbins, & Sahakian, 2008). The hBayesDM package currently contains three models for PRL tasks:

1.  prl_ewa (den Ouden et al., 2013)
  1 – Learning rate (0 < *φ* < 1)
  Experience decay (0 < *ρ* < 1)
  Inverse temperature (0 < *β* < 1)
2.  prl_fictitious (Gläscher et al., 2009)
  Learning rate (0 < *η* < 1)
  Indecision point (0 < *α* < 1)
  Inverse temperature (0 < *β* < 1)
3.  prl_rp (den Ouden et al., 2013)
  Reward learning rate (0 < *A*_*rew*_ < 1)
  Punishment learning rate (0 < *A*_*pun*_ < 1)
  Inverse temperature (0 < *β* < 1)

#### PRL: Parameter descriptions

All PRL models above contain learning rate parameters (refer to IGT: Parameter descriptions for details). The prl_rp model has separate learning rates for rewards (0 < *A*_*rew*_ < 1) and punishments (0 < *A*_*pun*_ < 1). In the prl_ewa model (Camerer & Ho, 1999), low and high values of *φ* reflect more weight on recent and on past outcomes, respectively. All PRL models also contain an inverse-temperature parameter (refer to DDT: Parameter descriptions for details).

The prl_ewa model proposed in den Ouden et al. (2013) contains a decay rate parameter (0 < *ρ* <). The experienced weight of the chosen option is decayed in proportion to *ρ*, and 1 is added to the weight on each trial. Thus, a higher value of *ρ* indicates slower decaying or updating of the experienced weight.

The prl_fictitious model contains an indecision point parameter (0 < *α* < 1). This point reflects a subject’s amount of bias or preference toward an option. High or low values for *α* indicate a greater or a lesser preference for one option over the other.

### Risk Aversion Task

The risk aversion (RA; Sokol-Hessner, Camerer, & Phelps, 2013; Sokol-Hessner et al., 2009) task is a description-based task (Hertwig, Barron, Weber, & Erev, 2004) in which the possible outcomes of all options and their probabilities are provided to subjects on each trial. In the RA task, subjects choose either a sure option with a guaranteed amount or a risky option (i.e., gamble) with possible gains and/or loss amounts. Subjects are asked to choose which option they prefer (or whether they want to accept the gamble) on each trial. In the RA task, subjects per form two cognitive regulation (*attend* and *regulate*) conditions in a within-subjects design: in the attend condition, subjects are asked to focus on each choice in isolation, whereas in the regulate condition, subjects are asked to emphasize choices in their greater context (see Sokol-Hessner et al., 2009, for the details). The data published in Sokol-Hessner et al. (2009) can be found using the following paths (these paths are also available in the RA model help files):

path_to_attend_data = **system.file**("extdata/ra_data_attend.txt", package="hBayesDM")

path_to_regulate_data = **system.file**("extdata/ra_data_reappraisal. txt", package="hBayesDM").

The hBayesDM package currently contains three models for the RA and similar (e.g., Tom, Fox, Trepel, & Poldrack, 2007) tasks:

1.  ra_prospect (Sokol-Hessner et al., 2009)
  Loss aversion (0 < *λ* < 5)
  Risk aversion (0 < *ρ* < 2)
  Inverse temperature (0<τ<∞)
2.  ra_noLA (no loss aversion [LA] parameter; for tasks that involve only gains)
  Risk aversion (0 < *ρ* < 2)
Inverse temperature (0<τ<∞)
3.  ra_noRA (no risk aversion [RA] parameter; see, e.g., Tom et al., 2007)
  Loss aversion (0 < *λ* < 2)
  Inverse temperature (0<τ<∞)

#### RA: Parameter descriptions

The ra_prospect model includes a loss aversion parameter (0 < *λ* < 5), a risk aversion parameter (0 < *ρ* < 2), and an inverse-temperature parameter (0<τ<∞). See DDT: Parameter descriptions for inverse temperature. The risk aversion and loss aversion parameters in the RA models are similar to those in the IGT models. However, in RA models they control the valuations of the possible choices under consideration, as opposed to the evaluation of outcomes after they are experienced (Rangel et al., 2008).

The ra_noLA and ra_noRA models are nested within the ra_prospect model, with either loss aversion (ra_noLA) or risk aversion (ra_noRA) set to 1.

### Two-Armed Bandit Task

Multi-armed bandit tasks or problems typically refer to situations in which gamblers decide which gamble or slot machine to play in order to maximize long-term gain. Many reinforcement-learning tasks and experience-based (Hertwig et al., 2004) tasks can be classified as bandit problems. In a typical two-armed bandit task, subjects are presented with two options (stimuli) on each trial. Feedback is given after a stimulus is chosen. Subjects are asked to maximize positive feedback as they make choices, and they are expected to learn stimulus–outcome contingencies from trial-by-trial experience. The hBayesDM package currently contains a simple model for a two-armed bandit task:

1.  bandit2arm_delta (Hertwig et al., 2004)
  Learning rate (0 < *A* < 1)
  Inverse temperature (0 < *τ* < 1)

#### Two-armed bandit: Parameter descriptions

The bandit2arm_delta model uses the Rescorla–Wagner rule (see IGT: Parameter descriptions) for updating the expected value of the chosen option, along with the softmax choice rule with an inverse temperature (see DDT: Parameter descriptions).

### The Ultimatum Game (Norm-Training)

The abilities to understand the social norms of an environment and to adaptively cope with those norms are critical for normal social functioning (Gu et al., 2015; Montague & Lohrenz, 2007). The ultimatum game (UG) is a widely used social decision-making task that examines how individuals respond to deviations from social norms and adapt to norms in a changing environment.

The UG involves two players: a proposer and a responder. On each trial, the proposer is given some amount of money to divide up amongst the two players. After deciding how to divide the money, an offer is made to the responder. The responder can either accept the offer (and the money is split as offered) or reject it (both players receive nothing). Previous studies have shown that the most common offer is approximately 50% of the total amount, and that “unfair” offers (<∼20% of the total amount) are often rejected, even though it is optimal to accept any offer (Güth, Schmittberger, & Schwarze, 1982; Sanfey, 2003; Thaler, 1988). A recent study examined the computational substrates of norm adjustment by using a norm-training UG in which subjects played the role of responder in a norm-changing environment (Xiang et al., 2013).

The UG has been used to investigate the social decision-making of individuals with ventromedial prefrontal (Gu et al., 2015; Koenigs et al., 2007) and insular cortex (Gu et al., 2015) lesions, as well as of patients with schizophrenia (Agay, Kron, Carmel, Mendlovic, & Levkovitz, 2008; Csukly, Polgár, Tombor, Réthelyi, & Kéri, 2011). The hBayesDM package currently contains two models for the UG (or norm-training UG) in which subjects play the role of responder:

1.  ug_bayes (Xiang et al., 2013)
  Envy (0 < *α* < 20)
  Guilt (0 < *β* < 10)
  Inverse temperature (0 < *τ* < 10)
2.  ug_delta (Gu et al., 2015)
  Envy (0 < *α* < 20)
  Inverse temperature (0 < *τ* < 10)
  Norm adaptation rate (0 < *ϵ* < 1)

#### UG: Parameter descriptions

The ug_bayes model assumes that the subject (responder) behaves like a *Bayesian ideal observer* (Knill & Pouget, 2004), so that the expected offer made by the proposer is updated in a Bayesian fashion. This is in contrast to the ug_delta model, which assumes that the subject (again the responder) updates the expected offer using a Rescorla–Wagner (delta) updating rule. Both the ug_bayes and ug_delta models contain envy (0 < *α* < 20) and inverse-temperature (0 < *τ* < 10; refer to DDT: Parameter descriptions for details) parameters. The envy parameter reflects sensitivity to norm prediction error (see below for the ug_bayes model), where higher or lower values indicate greater or lesser sensitivity, respectively. In the UG, prediction error reflects the difference between the expected and received offers.

In the ug_bayes model, the utility of an offer is adjusted by two norm prediction errors: (1) negative prediction errors, multiplied by an envy parameter (0 < *α* < 20), and (2) positive prediction errors, multiplied by a guilt parameter (0 < *β* < 10). Higher and lower values for envy (*α*) and guilt (*β*) reflect greater and lesser sensitivity to negative and positive norm prediction errors, respectively. The ug_delta model includes only the envy parameter (Gu et al., 2015).

## MATHEMATICAL FORMULATION OF HIERARCHICAL BAYESIAN MODELS

In this section, we briefly describe HBA for readers interested in HBA or Bayesian frameworks in general. Then we illustrate how we programmed our models using the Stan software package (Carpenter et al., 2016) and how we formulated hierarchical structures for various types of model parameters (see Performing Hierarchial Bayesian Analysis With Stan). Readers who are not interested in the mathematical details may skip the Performing Hierarchial Bayesian Analysis With Stan section.

Most computational models do not have closed-form solutions, so we need to estimate parameter values. Traditionally, parameters are estimated at the individual level with maximum likelihood estimation (MLE): getting point estimates that maximize the likelihood of data for each individual separately (e.g., Myung, 2003). However, the individual MLE estimates are often noisy and unreliable, especially when there are insufficient data, which is common in psychology or neuroscience experimental settings (cf. speeded choice-response time tasks). A group-level analysis (e.g., group-level MLE), which estimates a single set of parameters for the whole group of individuals, may generate more reliable estimates but inevitably ignores individual differences.

For parameter estimation, the hBayesDM package uses HBA, which is a branch of Bayesian statistics. We will briefly explain why hierarchical approaches such as HBA have advantages over traditional MLE methods. In Bayesian statistics, we assume prior beliefs (i.e., prior distributions) for the model parameters and update the priors into posterior distributions given the data (e.g., the trial-by-trial choices and outcomes) using Bayes’s rule. If Bayesian inference is performed individually for each individual *i*:

$$P\left(\Theta_{i}|D_{i}\right)=\frac{P\left(D_{i}|\Theta_{i}\right)P\left(\Theta_{i}\right)}{P\left(D_{i}\right)}=\frac{P\left(D_{i}|\Theta_{i}\right)P\left(\Theta_{i}\right)}{\int P\left(D_{i}|\Theta_{i\prime }\right)P\left(\Theta_{i\prime }\right)d\Theta_{i}\prime }$$

Here, Θ_*i*_ is the set of parameters of a model for individual *i* (e.g., $\Theta_{i}=\left\{\alpha_{i},\beta_{i},\gamma_{i},\cdots \;\right\}$), *D*_*i*_ is the data, *P*(*D*_*i*_|Θ_*i*_) is the *likelihood* (of the data given a set of parameters), *P*(*D*_*i*_) is called the *evidence* (of the data being generated by this model), and *P*(Θ_*i*_) and *P*(Θ_*i*_|*D*_*i*_) are the *prior* and *posterior* distributions of Θ_*i*_ , respectively.

**Figure 2.**
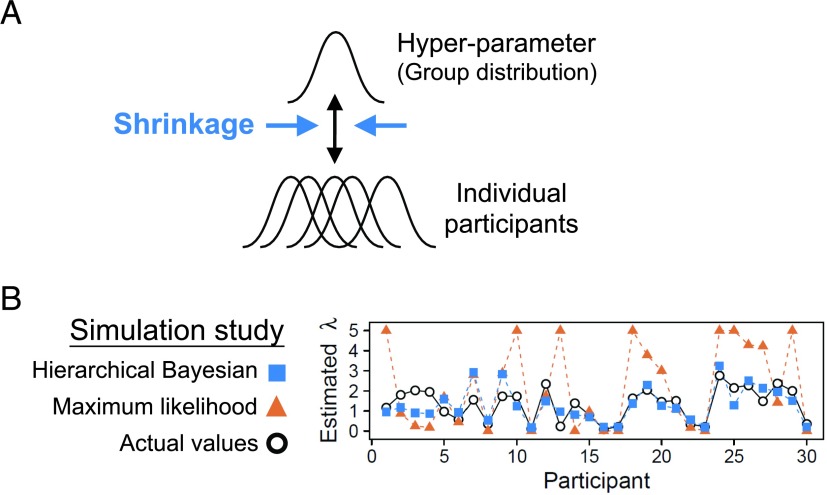
**(A) A schematic illustration of hierarchical Bayesian analysis (HBA). In this example, the individual parameters are assumed to come from a group (hyper)parameter. (B) Results of a parameter recovery study (Ahn et al., 2011) between HBA and maximum likelihood estimation.** Thirty subjects’ data from the Iowa gambling task were simulated using true parameters (black circles), and the parameters were estimated with hierarchical Bayesian analysis (blue squares = the individual posterior means) and individual maximum likelihood estimation (yellow triangles). The performance of the two approaches is shown for the loss aversion parameter (*λ*).

In HBA, hyperparameters are introduced in addition to the individual parameters, as is illustrated in Figure 2A (Gelman, Dunson, & Vehtari, 2013; Kruschke, 2014). If we set the hyperparameters as $\Phi =\left\{\mu_{\alpha },\mu_{\beta },\mu_{\gamma },\sigma_{\alpha },\sigma_{\beta },\sigma_{\gamma },\cdots \;\right\}$, with group-level normal means *μ*_(.)_ and standard deviations *σ*_(.)_, the joint posterior distribution $P\left(\Theta ,\Phi |D\right)$ is

$$P\left(\Theta ,\Phi |D\right)=\frac{P\left(D|\Theta ,\Phi \right)P\left(\Theta ,\Phi \right)}{P\left(D\right)}\propto P\left(D|\Theta \right)P\left(\Theta |\Phi \right)P(\Phi ).$$

The hierarchical structure of HBA leads to “shrinkage” effects (Gelman et al., 2013) in the individual estimates. Shrinkage effects, put simply, refer to when each individual’s estimates inform the group’s estimates, which in turn inform the estimates of all individuals. Consequently, the individual parameter estimates tend to be more stable and reliable, because commonalities among the individuals are captured and informed by the group tendencies (but see Future Directions section for the limitations and potential drawbacks of this approach). Such a hierarchical approach is particularly useful when the amount of information (e.g., the number of trials) from a single person is often too small to precisely estimate parameters at the individual level. A simulation study (Ahn, Krawitz, et al., 2011) empirically demonstrated that HBA outperforms individual MLE in parameter recovery (see Figure 2B), which suggests that parameter values estimated with HBA might more accurately reflect individual differences in underlying neurocognitive processes than do those estimated with individual MLE. Importantly, HBA provides full posterior distributions instead of point estimates; thus, it provides rich information about the parameters. HBA also makes it straightforward to make group comparisons in a Bayesian fashion (e.g., by comparing clinical and nonclinical groups; see section Compare models [and groups] for an example). Recent studies in cognitive and decision sciences further confirmed the validity and usefulness of HBA and other hierarchical approaches (e.g., Ahn et al., 2014; Guitart-Masip et al., 2012; Huys et al., 2011; Katahira, 2016; Lee, 2011; Raja Beharelle, Polania, Hare, & Ruff, 2015; Shiffrin, Lee, Kim, & Wagenmakers, 2008).

## PERFORMING HIERARCHICAL BAYESIAN ANALYSIS WITH STAN

In the hBayesDM package, posterior inference for all models is performed with a Markov chain Monte Carlo (MCMC) sampling scheme using the newly developed probabilistic programming language Stan (Carpenter et al., 2016) and its R instantiation, RStan (http://mc-stan.org/interfaces/rstan). Stan uses a specific MCMC sampler called Hamiltonian Monte Carlo (HMC) to perform sampling from the posterior distribution. During each iteration of HMC, derivatives of the density function, together with the auto-optimized Metropolis acceptance rate and step size and maximum steps, are utilized to find out the direction of the target posterior distribution (Carpenter et al., 2016). HMC offers more efficient sampling than conventional algorithms implemented in other software, such as BUGS (Lunn, Spiegelhalter, Thomas, & Best, 2009; Lunn, Thomas, Best, & Spiegelhalter, 2000) and JAGS (Plummer, 2003). Moreover, HMC works well even for complex models with high-dimensional model structures and highly correlated model parameters. A drawback of HMC is that it is not capable of directly sampling discrete parameters, because HMC uses derivatives of the density. However, one could marginalize the posterior density in order to obtain discrete outcomes. See the Stan reference manual (http://mc-stan.org/documentation/) and Kruschke (2014, chap. 14) for a comprehensive description of HMC and Stan. To learn more about the basic foundations of MCMC, see Krushcke (2014, chap. 7).

To use the hBayesDM package, users do not need to know how to program in Stan. However, for those interested in understanding our models and Stan in general, we briefly introduce the general structure of model specification in Stan, followed by the detailed hierarchical parameter declaration and optimizing approaches that are utilized in hBayesDM. Finally, we describe how we calculate log likelihood and model fits inside Stan models.

### General Structure of Stan Model Specification

Many useful features of BUGS were incorporated into Stan’s design; thus, Stan is similar to BUGS (or JAGS), and users who are familiar with BUGS will find Stan relatively easy to use (see the Stan reference manual, Appendix B; available at http://mc-stan.org/documentation/). There are six model blocks in the general structure of the Stan model specification, as listed below. Note that Stan implements sequential execution in its model specification, unlike BUGS and JAGS, in which the order of the code does not affect a model’s execution:

      … data {

      … read in external data …

      }


      transformed data {


      … pre-processing of data …


      }


      parameters {


       … parameters to be sampled by HMC…


      }


      transformed parameters {


      … pre-processing of parameters …


      }

      model {

      … statistical/cognitive model …

      }


      generated quantities {


      … post-processing of the model …


      }

Note that the data, parameters, and model blocks are mandatory in Stan, whereas the transformed data, transformed parameters, and generated quantities blocks are optional. Nonetheless, we typically use all of these optional blocks in hBayesDM, for different purposes: (1) We use the transformed data block to maintain a concise programming style and assign the initial values. (2) We implement noncentered parameterization (a.k.a. the “Matt trick”) in the transformed parameters block to optimize sampling and reduce autocorrelation be tween the group-level parameters in our hierarchical models. Details will be explained in the Optimizing Approaches in Stan section of this tutorial. (3) We include the generated quantities section to explicitly calculate the log-likelihood of the corresponding model and compute out-of-sample prediction accuracy (see Computing Log-Likelihood Inside Stan Models) for model comparison.

### Hierarchical Parameter Declaration in Stan

When declaring hierarchical parameters in hBayesDM with Stan, we assume that the individual parameters are drawn from group-level normal distributions. Normal and half-Cauchy distributions are used for the priors of the group-level normal means (*μ*_(.)_) and standard deviations (*σ*_(.)_), respectively. We employ flat (uniform) or weakly informative priors (Gelman et al., 2013) to minimize the influence of those priors on the posterior distributions when the sample sizes are small. We used standard normal priors for the group-level means (e.g., Lee, 2011; Shiffrin et al., 2008; Wetzels, Vandekerckhove, & Tuerlinckx, 2010), which also makes it easy to optimize Stan codes (see Optimizing Approaches to Stan). For the group-level standard deviations, we used half-Cauchy prior distributions, which tend to give sharper and more reasonable estimates than uniform or inverse-Gaussian prior distributions (Gelman, 2006). According to the range of the parameters of interest, we introduce four ways of declaring hierarchical parameters: unbounded parameters, positive parameters, parameters bounded between 0 and 1, and parameters bounded between 0 and an upper limit *U*.

For unbounded parameters (for illustration purposes, say *ξ* for a general individual parameter), we declare:       

$$\mu_{\xi }∼\text{Normal}(0,10)$$

      

$$\sigma_{\xi }∼\text{half-Cauchy}(0,5)$$

      

$$\xi ∼\text{Normal}(\mu_{\xi },\sigma_{\xi })$$

where *μ*_*ξ*_ (group mean parameter) is drawn from a wide normal distribution, *σ*_*ξ*_ (group standard deviation parameter) is drawn from a positive half-Cauchy distribution, and *ξ* is distributed as a normal distribution with a mean of *μ*_*ξ*_ and a standard deviation of *σ*_*ξ*_. Note that we use the wide normal distribution (weakly informative prior) so as to keep the prior bias minimum. Plus, the use of the positive half-Cauchy ensures that most of the density is between 0 and 10, while the HMC sampler is still able to visit beyond its upper bound, resulting in a soft constraint (Gelman et al., 2013).

For positive parameters (e.g., the effective size of reinforcements in the orthogonalized GNG task), we apply an exponential transformation to constrain an unbounded parameter to be greater than 0, such that the transformed prior is exclusively positive and avoids extreme values. Note that this results in a small bias toward zero. In hBayesDM, we define:

$$\mu_{\xi \prime }∼\text{Normal}(0,1)$$

$$\sigma_{\xi \prime }∼\text{half-Cauchy}(0,5)$$

$$\xi \prime ∼\text{Normal}(\mu_{\xi \prime },\sigma_{\xi \prime })$$

ξ=exp(ξ′)

For parameters that are bounded between 0 and 1 (e.g., learning rate), we use the inverse probit transformation (the cumulative distribution function of a unit normal distribution) to convert the unconstrained values into this range. In fact, given the mathematical relationship between the probability density function (pdf ) and the cumulative density function (cdf ) of the unit normal distribution, this transformation guarantees that the converted prior will be uniformly distributed between 0 and 1. Several studies have demonstrated the robustness and effectiveness of this transformation (e.g., Ahn et al., 2014; Wetzels et al., 2010). To effectively implement this, Stan provides a fast approximation of the inverse probit transformation (i.e., the Phi_approx function), which we adopted:

$$\mu_{\xi \prime }∼\text{Normal}(0,1)$$

$$\sigma_{\xi \prime }∼\text{half-Cauchy}(0,5)$$

$$\xi \prime ∼\text{Normal}(\mu_{\xi \prime },\sigma_{\xi \prime })$$

$$\xi ={\text{Probit}}^{-1}(\xi \prime )$$

For parameters that are bounded between 0 and an upper limit *U* (e.g., inverse softmax temperature, loss aversion in the RA task), we simply adapt the declaration rule for [0, 1] parameters and multiply it by the upper limit *U*. Likewise, the converted prior is distributed as a uniform distribution between 0 and *U*. If *U* is relatively small (less than ∼20), we use this approach instead of using a positive parameter (with an exponential transformation) in order to keep the prior bias minimal. When we use such an upper bound, the posterior fits are checked to ensure that the parameter estimates are not very close to the boundary. Formally, we declare:

$$\mu_{\xi \prime }∼\text{Normal}(0,1)$$

$$\sigma_{\xi \prime }∼\text{half-Cauchy}(0,5)$$

$$\xi \prime ∼\text{Normal}(\mu_{\xi \prime },\sigma_{\xi \prime })$$

$$\xi ={\text{Probit}}^{-1}(\xi \prime \cdot U)$$

As is shown above, we do not employ truncated sampling in declaring the hierarchical parameters because hard constraints (e.g., *ξ* ∼ Normal(0,1)T[0,*U*]) may harm the HMC sampling algorithm and return poorly converging posterior distributions (Carpenter et al., 2016). If users want to build their own hierarchical Bayesian models for their research, they can refer to our practice of standardizing the parameter declarations.

### Optimizing Approaches in Stan

Hierarchical models often suffer from highly correlated group-level parameters in their posterior distributions, creating challenges in terms of model convergence and estimation time (Gelman et al., 2013; Kruschke, 2014). To address these challenges, we practice reparameterization and vectorization in order to optimize the model specification in hBayesDM.

A Normal(*μ*,*σ*) distribution, like other distributions in the location–scale distribution family, can be reparameterized to be sampled from a unit normal distribution that is multiplied by the scale parameter *σ* and then shifted with the location parameter *μ*. Formally,

$$\xi ∼\text{Normal}(\mu_{\xi },\sigma_{\xi })$$

is mathematically equivalent to

ξ′∼Normal(0,1),

$$\xi ∼\text{Normal}\mu_{\xi }+\xi \prime \cdot \sigma_{\xi }).$$

Such transformation is referred to as *noncentered parameterization* (a.k.a. the “Matt trick”) by the Stan Development Team (2016), and it effectively reduces the dependence between *μ*_*ξ*_, *ξ*, and *σ*_*ξ*_ and increases the effective sample size.

In addition to reparameterization, we use vectorization to improve our MCMC sampling. For example, suppose that one experiment consists of *N* participants; then, its individual-level parameter *ξ* is an *N*-dimensional vector. Instead of declaring *ξ* as

for(nin1…N),

$$\xi_{\left[n\right]}∼\text{Normal}(\mu_{\xi },\sigma_{\xi }),$$

we vectorize it as

$$\xi ∼\text{Normal}(\mu_{\xi },\sigma_{\xi })$$

to make full use of Stan’s vectorization of all sampling statements. As a rule of thumb, one may want to use vectorization for as long as this is possible. All of hBayesDM’s models that implement both reparameterization and vectorization can be found in the directory …\R\R-x.x.x\library\hBayesDM\stan, or the path can be retrieved by calling the following R command: file.path(.libPaths(), "hBayesDM",
                        "stan"). Those interested in more details about optimizing Stan models can read the Stan reference manual (http://mc-stan.org/documentation/, chapter on “Optimizing Stan Code”).

### Computing Log-Likelihood Inside Stan Models

The hBayesDM package provides two model performance indices: the leave-one-out infor mation criterion (LOOIC) and the widely applicable information criterion (WAIC). We follow Vehtari, Gelman, and Gabry (2016) in computing and monitoring Stan’s pointwise log-likelihood in the generated quantities block. The generated quantities block serves as the postprocessing of the model, with its commands being executed only after the HMC sampling. Therefore, it does not significantly increase the time required for Bayesian inference. The generated quantities block is particularly useful when users intend to monitor pointwise log-likelihood (Vehtari et al., 2016), reproduce predictive values or obtain internal model variables. Practically, we initialize the pointwise log-likelihood to be 0 for each participant, then we repeat the same model of the “model” block in the generated quantities block, except we replace the sampling statement with the explicit computation of pointwise log-likelihood. Please be aware that in many RLDM tasks (especially RL tasks), choices on one trial are dependent on those on other trials. Therefore, instead of gathering the trial-by-trial log-likelihood, we sum them over per participant and obtain the pointwise log-likelihood at the participant level. Below is the pseudocode as an example of what is described above:

      model {

      …

       for (i in 1:N) {

        for (t in 1:T) {

         Choice[i, t] ∼ categorical_logit(ev);

      …

      }

      Generated quantities {

      …

       for (i in 1:N) {

        log_lik[i] = 0;

        for (t in 1:T) {

         log_lik[i]=log_lik[i] + categorical_logit_lpmf(Choice[i, t] | ev);

      …

      }

Once we have the pointwise log-likelihood per participant, it is straightforward to compute both LOOIC and WAIC (Vehtari et al., 2016). Both LOOIC and WAIC provide estimates of the out-of-sample predictive accuracy in a fully Bayesian way, which samples new participants from the hierarchical group, generates new data from those new participants, and evaluates how well a model makes predictions about the new dataset. What makes LOOIC and WAIC more reliable than the Akaike information criterion (AIC; Akaike, 1987; Bozdogan, 1987) and the deviance information criterion (DIC; Spiegelhalter, Best, Carlin, & van der Linde, 2002) is that both LOOIC and WAIC use the pointwise log-likelihood of the full Bayesian posterior distribution, whereas AIC and DIC use only point estimates to calculate the model evidence. We used the functions included in the loo package to (Vehtari et al., 2016) generate the LOOIC and WAIC values. Both LOOIC and WAIC are on the information criterion scale; thus, lower values of LOOIC or WAIC indicate better out-of-sample prediction accuracy of the candidate model.

## STEP-BY-STEP TUTORIALS FOR THE hBayesDM PACKAGE

### Installing hBayesDM: Prerequisites

Before installing hBayesDM, it is necessary to have up-to-date versions of R (version 3.3.2 or later is recommended) and RStan on your machine. RStudio (www.rstudio.com) is not required but is strongly recommended. Typically, RStan can be installed just by entering the following command into the R console:

**install.packages**("rstan",
                                    dependencies=TRUE)

For Windows, it is necessary to install Rtools before installing RStan. Instructions for installing Rtools on a Windows machine can be found at this link (https://github.com/stan-dev/rstan/wiki/Install-Rtools-for-Windows). After RStan (as well as Rtools, for Windows users) is installed, it is recommended to restart R (or RStudio) and test the installation before moving on to install hBayesDM. This can be accomplished by trying to fit the “Eight Schools” example that is provided on RStan’s Getting Started page (https://github.com/stan-dev/rstan/wiki/RStan-Getting-Started).

### Installing hBayesDM

The hBayesDM package is available from the Comprehensive R Archive Network (CRAN) and GitHub (https://github.com/CCS-Lab/hBayesDM). To install hBayesDM from CRAN, use the following call:

**install.packages**("hBayesDM",
                                    dependencies=TRUE)

For Mac or Linux computers, we recommend installing the latest version of hBayesDM from GitHub:


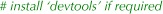


**if** (!**require**(devtools)) install.packages("devtools")

devtools::**install_github**("CCS-Lab/hBayesDM")

Stan models installed through GitHub are precompiled, so the models will run immediately without additional compilation time. As of March 2017, this feature is not available for Windows computers yet.

### How to Use hBayesDM: Navigating

After hBayesDM has been installed correctly, the package must be loaded into the current environment. Users will be able to access all the functions that are included in the package after hBayesDM is loaded. To load hBayesDM, use the following command:

**library**(hBayesDM)

After loading the package, users should see a message that displays the version number of the current hBayesDM install. For a list of all the models available in the package, one can refer to the package help files by using the following command:

**?**hBayesDM

This will bring up a help file that contains a general description of the package along with a list of all RLDM tasks and models that can be fit with hBayesDM. One can follow the links provided in the help file to find in-depth documentation describing how to fit each model.

### How to Use hBayesDM: Model Fitting

**Figure 3.**
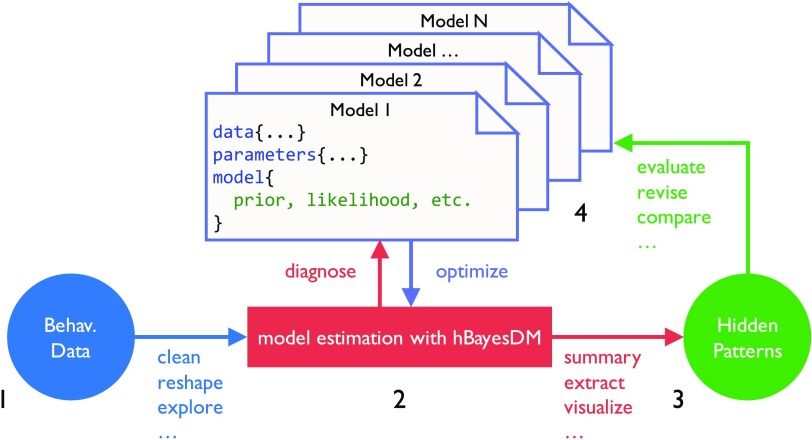
**Pipeline for performing computational modeling with hBayesDM.** Four steps are involved in hierarchical Bayesian analysis (HBA): (1) preparing the data, (2) fitting the candidate models, (3) extracting and visualizing the parameters and/or variables, and (4) model comparison (see the text for details).

The conceptual framework of computational modeling and the four steps of doing HBA with hBayesDM are illustrated graphically in Figure 3. These steps are described in further detail below. To exemplify these steps, the four models of the orthogonalized GNG task will be fit and compared using the hBayesDM package. As a reminder, users can refer to the help file for any model to learn how to run a real-data example. Also, commands and input arguments for running or evaluating a model are very similar or the same for all models. Thus, if users learn how to run one model, they can also easily run other models.

#### Prepare the data

To begin, all subjects’ data (for the current analysis) should be combined into a single text (*.txt) file, in which rows represent trial-by-trial observations and columns represent the variables of interest. The first row of the text file must contain the column headers (i.e., the names) of the variables of interest.

Subjects’ data must contain variable headers that are consistent with the column names specified in the model help file (see Table 1). For example, in the orthogonalized GNG task, the columns should be labeled “subjID,” “cue,” “keyPressed,” and “outcome,” where “subjID” is a subject-specific identifier, “cue” is a nominal integer specifying the cue shown on the given trial, “keyPressed” is a binary value representing whether a key was (1) or was not (0) pressed on the given trial, and “outcome” represents a positive (1), negative (–1), or neutral (0) outcome on the given trial. The text file may also contain other data/column headers, but only the aforementioned variables will be used for the modeling analysis. All of the information above for each model can be found in the package help files, which can be accessed with R’s help command (e.g., for the orthogonalized GNG Model 1, **?**gng_m1). Across all the models implemented in hBayesDM, the number of trials within the data file is allowed to vary across subjects, but the file should contain no missing (N/A) data. If some trials do contain N/A data (e.g., outcome=NA), remove these trials before continuing. If trials containing N/A data are not removed prior to the model fitting, they will be removed automatically and the user will receive a warning message.

Sample data can be retrieved from the package folder with the R command shown below. Note that the file name of sample (example) data for a given task is **taskName_exampleData.txt** (e.g., dd_exampleData.txt, igt_exampleData.txt, or gng_exampleData.txt):

dataPath = **system.file**("extdata/gng_exampleData.txt", package="hBayesDM")

gng_data = **read.table**(dataPath,
                                        header=TRUE)

If data are downloaded from an external source to "/home/user1/Downloads", the user may specify the path using a character string like the one below:

dataPath = "/home/user1/Downloads/gng_exampleData.txt"

#### Fit candidate models

Since hBayesDM uses MCMC sampling to generate posterior distributions, many arguments may be passed to Stan through the model functions in order to fine-tune the sampling behavior. Some arguments can also be used for user convenience. Table 2 shows the arguments that are common to all model functions in hBayesDM. Note that in the table an asterisk (*) denotes an argument that may unpredictably change the computation time and/or sampling behavior of the MCMC chains (Hoffman & Gelman, 2014). For this reason, it is advised that only advanced users alter the default values of these arguments.

**Table 2.** List of input arguments common to all model functions in the hBayesDM package

**Argument**	**Required From User**	**Description**
data	Yes (default = “choose”)	Full path to text file containing data to be used for analysis
niter	No (default = 2,000 or higher)	Number of (accepted) samples to be generated by Stan’s HMC sampler
nwarmup	No (default = 1,000)	Number of (accepted) samples to be discarded from the beginning of the sampling procedure
ncore	No (default = 1)	Number of CPUs to use for parallel computing
nchain	No (default = 4)	Number of MCMC chains to run
nthin	No (default = 1)	Every *n*th (accepted) sample from the sampling procedure will be saved. All other samples will be discarded
inits	No (default = “random”)	Initial values for the HMC sampler
indPars	No (default = “mean”)	How to summarize the parameters upon completion (mean, median, or mode)
saveDir	No (default = NULL)	Path to directory where hBayesDM object should be saved upon completion
email	No (default = NULL)	Email address that will be sent a message upon sampling completion
modelRegressor	No (default = FALSE)	Exporting model-based regressors? TRUE or FALSE
*adapt_delta	No (default = 0.95)	Acceptance probability of the HMC sampler
*stepsize	No (default = 1)	Size of each leapfrog step that the MCMC sampler can take on each new iteration.
*max_treedepth	No (default = 10)	Number of leapfrog steps that the MCMC sampler can take on each new iteration.

Below, the gng_m1 model is fit using the sample data that come with the package. The command indicates that three MCMC chains are to be run and three cores are to be used for parallel computing. Note that parallel computing is only useful for multiple chains; it is common to use one core per chain, to maximize sampling efficiency. If "example" is entered as an argument for data, hBayesDM will use the sample data for the task. Convenience arguments such as saveDir can be used to save the resulting model output to a local directory. This is useful when the model fitting is expected to take a long period of time and users want to ensure that the data are saved. Also, the email argument allows users to be notified by an e-mail message upon the completion of model fitting.

output1 = **gng_m1**("example",niter=2000,nwarmup=1000,nchain=4,

      ncore=4, saveDir="/data/Models",

      email="email@gmail.com")

A model function has default values for all arguments except data, so the command above is equivalent (aside from the saveDir and email arguments) to the more concise call below:

output1 = **gng_m1**("example",
                                        nchain=4, ncore=4)

If the data argument is left blank, a file browser window will appear, allowing the user to manually select the text file with their file browser. The default input arguments for each model were selected on the basis of our experience with the sampling behavior of each model with respect to the data we have access to. For each model being fitted, niter and nwarmup values (and control parameters, for advanced users) might need to be experimented with to ensure that the target posterior distributions will converge. Later sections will discuss convergence in more detail.

Executing any model function command in hBayesDM will generate messages for the user within the R console, exemplified by Figure 4A. It will take up to approximately 3 min (with the gng_m1 model and "example" data) for the model fitting to complete. Note that you may get warning messages about “numerical problems” or that there are a certain number of “divergent transitions after warm-up.” When we check our models with example datasets, warning messages appear mostly at the beginning of the warm-up period, and very few divergent transitions occur after warm-up. In such cases, the warnings can be ignored. For a technical description of these (and other) sampling issues, see Appendix D of the Stan Reference Manual. When the model fitting is complete, the R console will print the message in Figure 4B. The output data will be stored in output1, a class hBayesDM object containing a list with the six following elements:

1.  model:
  Name of the fitted model (i.e., output1$model is "gng_m1")
2.  allIndPars:
  Summary of individual subjects’ parameters (default: posterior *mean values of individual parameters*). Users can also choose to use the posterior *median* or *mode* in the model function command (e.g., indPars="mode"). See Figure 4C to view the values of allIndPars for gng_m1, printed to the R console.
3.  parVals:
  Posterior MCMC samples for all parameters. Note that hyper (group) posterior mean parameters are indicated by mu_PARAMETER (e.g., mu_xi, mu_ep, mu_rho). These values are extracted from the fit element with RStan’s extract() function.
4.  fit:
  An rstan object is the output of RStan’s stan() function. If users would like to use Rstan commands, the commands should be performed on this object. See Figure 4D for a summary of fit printed to the R console.
5.  rawdata:
  Raw trial-by-trial data used for HBA. The raw data are provided in the output to allow users to easily access them and compare the trial-by-trial model-based regressors (e.g., prediction errors) with the choice data.
6.  modelRegressor (optional):
  Trial-by-trial model-based regressors, such as prediction errors, the value of the chosen option, and so forth. For each model, we preselected appropriate model-based regressors. Users can refer to the package help files for the details. Currently (version 0.3.0), this feature is available only for the orthogonalized GNG task.
  **Figure 4.**
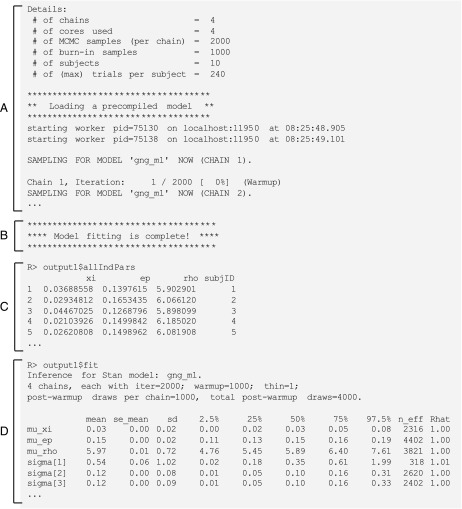
**Outputs of model fitting and model summary.** (A) Sample message displayed in the R console after a model function is called. Here, the Details section of the output shows information relevant to both the arguments passed to the function and the data specified by the user. The console also shows the progression of the MCMC sampling. (B) Upon completion of the model fitting, a message is presented to the user. (C, D) Displays from which users can retrieve summary statistics of the (C) individual model parameters and (D) Stan model fits (for the Stan fit object stored as output1).

#### Plot model parameters

It is important to both visually and quantitatively diagnose MCMC performance (i.e., visually check whether the MCMC samples are well mixed and converge to stationary distributions). For visual diagnostics of hyper (group) parameters, users can call plot.hBayesDM() or just plot(), which searches for an extension function that contains the class name. The class of any hBayesDM output is hBayesDM. For a quantitative check on convergence, the Gelman–Rubin convergence diagnostic (Gelman & Rubin, 1992) for each parameter is computed by RStan and stored in the fit element of the hBayesDM model output. These values may be seen in Figure 4D, where $\hat{R}$ (Rhat) is the Gelman–Rubin index used to assess the convergence of the MCMC samples. $\hat{R}$ values close to 1.00 indicate that the MCMC chains have converged to stationary target distributions. Values greater than 1.1 are typically considered to represent inadequate convergence. For all models included in hBayesDM, the $\hat{R}$ values are 1.00 for most parameters, or at most 1.04 when tested on the example datasets.

**Figure 5.**
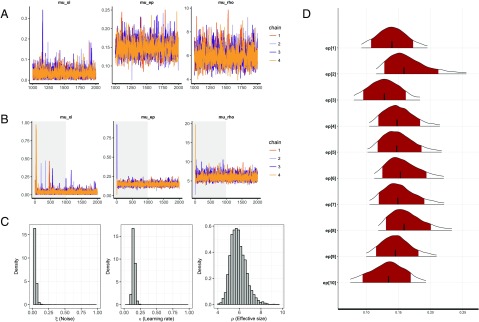
**Visualization of the parameters of the gng_m1 model.** (A) Trace plots for the group-level (hyper)parameters of the gng_m1 model. The three chains show excellent mixing, suggesting that they have converged to their target distributions. (B) The same trace plots as in panel A; however, these versions also include the warm-up (burn-in) samples, highlighted by the gray back ground shading. (C) Posterior distributions of the group-level (hyper)parameters. (D) Individual-level posterior distributions. The red shading and tailed white areas represent the 80% and 95% kernel density estimates, respectively. Note that all plots above were generated directly from hBayesDM and RStan functions, with no further modifications.

Users can also use trace plots to visually check the MCMC samples. The command shown below (with the font size for the plot set to 11) shows how to use the plot() command to create trace plots of hyper (group) parameters (see Figure 5A for an example):

**plot**(output1, type="trace",
                                        fontSize=11)

The trace plots indicate that the MCMC samples are indeed well mixed and have converged, which is consistent with their $\hat{R}$ values. Note that the plots in Figure 5A exclude burn-in samples. Users can include burn-in (warm-up) MCMC samples to better understand the sampling behavior, if necessary. The following function call produces the plot in Figure 5B, which includes burn-in samples:

**plot**(output1, type="trace",
                                        inc_warmup=T)

Users can also plot the posterior distributions of the hyper (group) parameters with the default plot() function by not specifying the type argument. The following function call produces the plot in Figure 5C:

**plot**(output1)

To visualize the individual parameters, users can use the plotInd() command. The following call plots each individual’s *ϵ* (learning rate) parameter (see Figure 5D):

**plotInd**(output1,
                                        "ep")

#### Compare models (and groups)

To compare multiple models using LOOIC or WAIC values, the first step is to fit all models in the same manner as the gng_m1 example above. The following commands will fit the rest of the orthogonalized GNG models available within hBayesDM:

output2 = **gng_m2**("example",
                                        nchain=4, ncore=4)

output3 = **gng_m3**("example",
                                        nchain=4, ncore=4)

output4 = **gng_m4**("example",
                                        nchain=4, ncore=4)

Note that each model should be checked for convergence in the same manner as gng_m1. If for any reason a model fails to converge, refit the model after model diagnostics (see Improving sampling performance in hBayesDM) or exclude the model from the model comparisons.

Next, users can assess the model fits using the printFit() command, which is a convenient way to summarize the LOOIC and WAIC of all considered models. Assuming that all four models’ outputs are named output1 (gng_m1), output2 (gng_m2), output3 (gng_m3), and output4 (gng_m4), their model fits can be simultaneously summarized by the following command, the results of which are illustrated in 


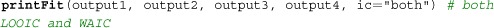


**Figure 6.**
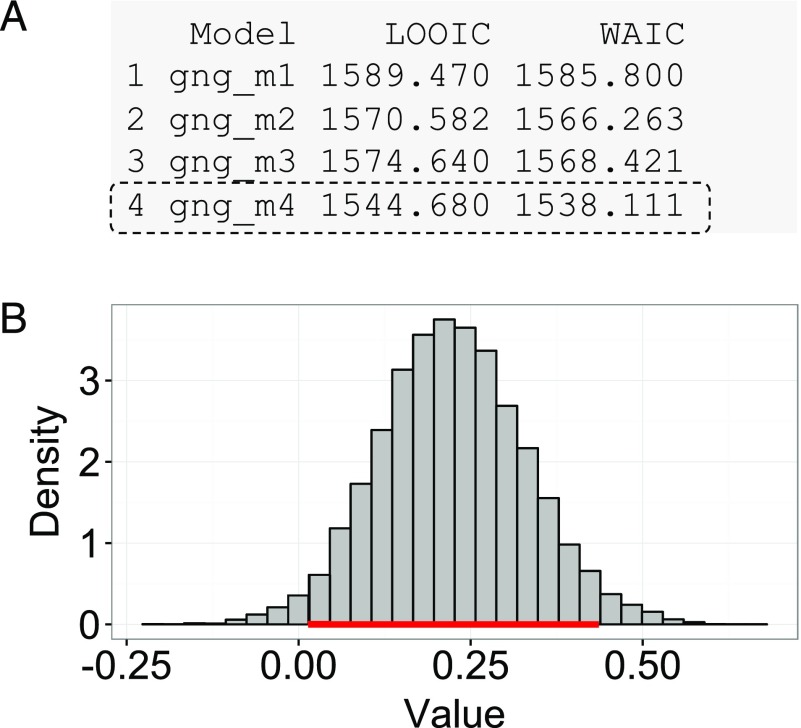
**Bayesian model selection and group comparison.** (A) Sample output of the printFit() command, which prints model performance indices (LOOIC and WAIC) for competing model(s). The resulting table shows the name of each model, followed by their LOOIC and WAIC values. Lower LOOIC and WAIC values correspond to better model performance. Here, gng_m4 (highlighted with a dashed box) has the lowest values. (B) Results from the plotHDI() function, showing the 95% highest density interval (HDI) of the posterior distribution difference between two group parameters. The red bar indicates the 95% HDI.

By default, the printFit function uses LOOIC, which is preferable to WAIC when there are influential observations (Vehtari et al., 2016). Lower LOOIC or WAIC values indicate better model performance; thus, Model 4 has the best LOOIC and WAIC, as compared to all other models. Users interested in more detailed information, including standard errors and the expected log pointwise predictive density (elpd), can use the extract_ic() function (e.g., extract_ic(output3)) to extract this information. Note that the extract_ic() function can be used only for a single model output, unlike printFit().

Other model comparison methods exist, including the simulation method (a.k.a. absolute model performance; Ahn et al., 2008; Ahn et al., 2014; Guitart-Masip et al., 2012; Steingroever, Wetzels, & Wagenmakers, 2014), parameter recovery (Ahn, Krawitz, et al., 2011; Ahn et al., 2014), and the generalization criterion (Ahn et al., 2008; Busemeyer & Wang, 2000). Models that show the best goodness of fit may not perform well according to other indices (e.g., Ahn et al., 2014), so it is recommended that researchers use multiple model comparison methods if this is at all possible.

#### Group comparisons

Having selected the best-fitting model, users may want to use that model to compare the parameter estimates of different populations. With a hierarchical Bayesian framework, users can compare the model parameters of multiple groups or within-subjects conditions in fully Bayesian ways (e.g., Ahn et al., 2014; Chan et al., 2014; Fridberg, Ahn, Kim, Bishara, & Stout, 2010; Kruschke, 2014; Vassileva et al., 2013). The (posterior) distributions show the uncertainty in the estimated parameters, and we can use the posterior highest density interval (HDI) to summarize the uncertainty. The *95% HDI* refers to “the span of values that are most credible and cover 95% of the posterior distribution” (Kruschke, 2014). To examine the difference in a particular parameter between two groups, we can calculate the difference between the hyperdistributions across the groups and examine the credible interval of this difference (i.e., its 95% HDI; Kruschke, 2010, 2011). Note that this is different from testing a null hypothesis (e.g., whether or not two groups are the same on the parameter of interest), for which Bayesian hypothesis testing (e.g., the Bayes factor; Kass & Raftery, 1995; Myung & Pitt, 1997; Wagenmakers, 2007) or a region of practical equivalence (ROPE) around the null value should be used instead (Kruschke, 2011, 2014).

As an example, we compare two groups’ model parameters in a Bayesian fashion. First, prepare each group’s data as separate text files:

data_group1 = " ∼/Project_folder/gng_data_group1.txt"

data_group2 = "∼/Project_folder/gng_data_group2.txt"

Here, gng_data_group1.txt and gng_data_group2.txt contain all the data for the Group 1 subjects and the Group 2 subjects, respectively. Next, the model is fit in the same manner as before for each group separately. We recommend using the same numbers of chains and MCMC samples for each group:

output_group1 = **gng_m4**(data_group1,nchain=4, ncore=4)

output_group2 = **gng_m4**(data_group2, nchain=4, ncore=4)

Make sure to check whether the MCMC samples are well mixed and converge to stationary distributions (see Plot model parameters). Next, compute the difference between the hyper (group) parameters of interest by making a simple subtraction. For example, if we want to compare the Pavlovian bias parameters (*π*) across the two groups:

diffDist = output_group1$parVals$mu_pi - output_group2$parVals$mu_pi

The command above subtracts the mu_pi parameter of Group 2 from that of Group 1. Note that these parameter values are stored within the parVals element of an hBayesDM object. To generate the credible interval of the difference between the groups, users can use the following command, which will print the 95% HDI to the R console:

**HDIofMCMC**(diffDist)

Users can also visually inspect the 95% HDI with the following command (the 95% HDI is also printed to the R console in response to the command):

**plotHDI**(diffDist)

Figure 6B shows the result of the plotHDI() command above. The red bar along the bottom of the plot encompasses the 95% HDI.

#### Improving sampling performance in hBayesDM

When chains fail to converge (e.g., $\hat{R}$ > 1.10 or the MCMC chains are poorly mixed when visually inspected), users are recommended to use different starting values for multiple chains or to modify several HMC sampling parameters to improve the performance. Users can set inits="fixed" to use initial values that are provided by the developers (e.g., output=**gng_m4**("example",
                            inits="fixed")) or can provide their own starting values (e.g., inits=c(0.1, 0.2, 10) for gng_m1).

With respect to the HMC sampling parameters, though a model’s performance may be model- and parameter-specific, we provide a general approach for users to experiment with. Three parameters are relevant for sampling performance: the Metropolis acceptance rate (*δ*, default = 0.95), the initial HMC step size (*ε*, default = 1.0), and the maximum HMC steps per iteration (*L*; i.e., the maximum tree depth, default = 10). We refer readers to the Stan help file(?stan) for more details. With default sampling parameters and sample datasets, all models implemented in the hBayesDM package showed excellent convergence and mixing of the MCMC chains. However, if users notice any signs of poor convergence or mixing, we suggest that they increase *δ*, decrease *ε*, and/or increase *L*. The adjustment in hBayesDM is illustrated below (taking gng_m1 as an example):

output1=
                                        **gng_m1**("example",nchain=4,
                                        ncore=4, adapt_delta=0.99, stepsize=0.5,
                                        max_treedepth=20)

Be aware that such an adjustment might dramatically increase the model estimation time and does not necessarily guarantee improved sampling performance. The failure of an adjusted model estimate might further suggest that such a model is not suitable for the current dataset, and that one may need to consider using alternative models to fit the data. If users encounter a problem and would like to seek help from the hBayesDM developers, they can ask questions to our mailing list (https://groups.google.com/forum/#!forum/hbayesdm-users).

### Extracting Trial-by-Trial Regressors for Model-Based fMRI/EEG Analysis

In model-based fMRI or EEG (Mars et al., 2008; see, e.g., O’Doherty et al., 2007), model-based time series of a latent cognitive process are generated by computational models, and then the time-series data are regressed again using fMRI or EEG data. This model-based neuroimaging approach has been particularly popular in cognitive neuroscience (e.g., Ahn, Krawits, et al., 2011; Behrens, Woolrich, Walton, & Rushworth, 2007; Daw et al., 2006; Gläscher, Daw, Dayan, & Doherty, 2010; Gläscher et al., 2009; Hampton et al., 2006; Iglesias et al., 2013; Kable & Glimcher, 2007; O’Doherty, Critchley, Deichmann, & Dolan, 2003; O’Doherty et al., 2007; Xiang et al., 2013) to identify brain regions that presumably implement a cognitive process of interest.

**Figure 7.**
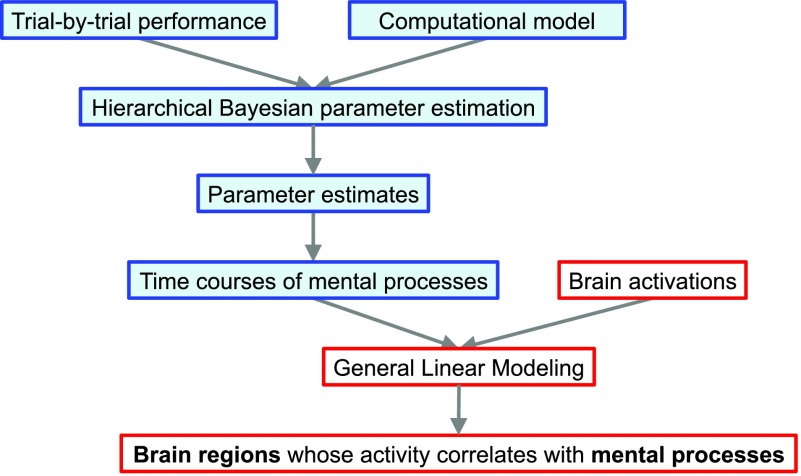
**Steps of model-based fMRI.** With the hBayesDM package, users can perform the steps highlighted in blue. Users need to use a neuroimaging tool of their choice (e.g., SPM) to perform the steps highlighted in red.

The hBayesDM package allows users to extract various model-based regressors that can be used for model-based fMRI or EEG analysis (see Figure 7). All model-based regressors are contained in the modelRegressor element. Note that in the current version (0.3.0), only the orthogonalized GNG task provides model-based regressors. The hBayesDM package provides the following model-based regressors, and users can convolve these trial-by-trial data with a hemodynamic response function using their favorite package (e.g., in the SPM package [www.fil.ion.ucl.ac.uk/spm/], users can use the parametric modulation command with a model-based regressor):

1.  Stimulus value: *V*_*t*_(*s*_*t*_) (stored as SV; available in gng_m3 and gng_m4)
2.  Action value: *Q*_*t*_(*go*) (stored as Qgo) and *Q*_*t*_(*NoGo*) (stored as Qnogo)
3.  Action weight: *W*_*t*_(*go*) (stored as Wgo) and *W*_*t*_(*NoGo*) (stored as Wnogo)

For example, to retrieve the stimulus value (= *V*_*t*_(*s*_*t*_)) of Group 1 in the previous example (the output is saved as output_group1), type:

      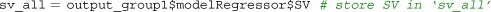


Here, sv_all is an array (the number of rows = number of subjects, the number of columns = number of trials). Similarly, to retrieve action weight values (*W*_*t*_(*go*) and (*W*_*t*_(*NoGo*)), type:

      

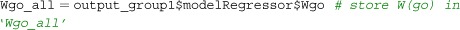


      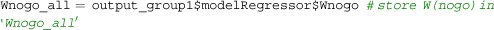


Users can use these values for each subject to perform model-based fMRI analysis with their favorite neuroimaging package (O’Doherty et al., 2007). Once the model-based regressors are entered as parametric modulators in a generalized linear model (GLM), neuroimaging tools convolve the regressors with the hemodynamic response function and construct a new GLM. For step-by-step tutorials for model-based fMRI, see the following online documents: www.translationalneuromodeling.org/uploads/Mathys2016_SPMZurich_ModelBasedfMRI.pdf; www.translationalneuromodeling.org/uploads/DiaconescuAndreea_Model-based_fMRI.pdf; www.srndna.org/conference2015/files/2014/11/SRNDNA_RL_Modeling_wkshp2.pdf.

## FUTURE DIRECTIONS

In the current version, the hBayesDM package selectively implements seven commonly used RLDM tasks and their models, but we plan to expand the list of tasks and models so that the hBayesDM can handle an extensive list of RLDM tasks. Latent model-based regressors are available only for a single task, but they will be available for more tasks in a future release of the hBayesDM package. We also plan to develop a graphical user interface using the Shiny framework (https://shiny.rstudio.com/), so that users can select a dataset and run models without any R programming knowledge.

The hBayesDM package is useful for researchers across all levels of experience, including experts in computational modeling—hBayesDM systematically implements HBA of various computational models, and we find it useful and easier to build new models based on the existing framework. We welcome collaboration and others’ contributions to the package. We plan to release a more detailed tutorial on how to modify existing codes and build new models based on our framework.

In our HBA framework, it is assumed that there is a single hypergroup across all subjects. Although this assumption allows more precise estimates with a modest number of subjects (Ahn, Krawitz, et al., 2011; Katahira, 2016), it might be invalid with a large (e.g., ˜1,000) number of subjects (Ahn & Busemeyer, 2016; Ratcliff & Childers, 2015). Bayesian hierarchical mixture approaches (Bartlema, Lee, Wetzels, & Vanpaemel, 2014) or HBA on subgroups first clustered by behavioral indices might be alternative solutions when a large number of samples need to be fitted.

In conclusion, the hBayesDM package will allow researchers with a minimal quantitative background to do cutting-edge hierarchical modeling of a variety of RLDM tasks. With hBayesDM, researchers can also easily generate the model-based regressors required for model-based fMRI/EEG analysis. It is our expectation that the hBayesDM package will contribute to the dissemination of computational modeling and computational psychiatric research for researchers in various fields, including mental health.

## AUTHOR CONTRIBUTIONS

W.-Y.A. conceived and designed the project. W.-Y.A., N.H., and L.Z. programmed codes for the hierarchical Bayesian modeling. N.H. built an R package and wrote the help files. W.-Y.A., N.H., and L.Z. wrote the article.

## ACKNOWLEDGMENTS

W.-Y.A. programmed prototypes for several of the tasks while he was advised by Jerome Busemeyer (for the Iowa gambling task) or P. Read Montague/Peter Dayan (for the orthogonalized go/no-go, two-step, and risk aversion tasks, as well as the ultimatum game). We thank them for their guidance and the resources provided to W.-Y.A. We thank Peter Sokol-Hessner for sharing data published in Sokol-Hessner et al. (2009). L.Z. was partially supported by the German Research Foundation (DFG GRK 1247) and the Bernstein Computational Neuroscience Program of the German Federal Ministry of Education and Research (Grant 01GQ1006).
